# Management of splenic “incidentalomas” found on ultrasound and computed tomography

**DOI:** 10.1186/1470-7330-15-S1-O11

**Published:** 2015-10-02

**Authors:** Richard M Gore, Jacob S Ecanow

**Affiliations:** 1 Department of Radiology, NorthShore University HealthSystem, Evanston, IL. 60201, USA; 2 University of Chicago, Chicago, IL, 60637, USA

## Introduction

Splenic “incidentalomas” are focal lesions of the spleen that are discovered on imaging studies that were performed for the evaluation of non-spleen related pathology. These lesions are frequently encountered on CT and ultrasound examinations. Most incidental focal splenic lesions are benign, however a systematic approach to evaluating these lesions is important, because many benign and malignant splenic masses share common imaging features [1,2].

## The odds

Small studies report the prevalence of incidental splenic lesions on CT scans performed on trauma patients to be less than 1.5% [2]. Metastases and lymphoma are the two most common splenic malignancies, and although both are very rarely seen in the setting of an incidental finding. There are only a few dozen reports of isolated splenic metastases in the literature, so incidental splenic lesions without any evidence of metastases elsewhere are unlikely to be metastatic [3]. Similarly, most splenic lymphomas are seen in patients with systemic disease, and evidence of lymphoma can be found elsewhere on the scan.

## Morphologic clues

Asymptomatic simple cysts are almost always benign; these are most commonly false cysts due to prior hematoma or infarct, as well as true congenital epidermoid cysts and pancreatic pseudocysts. Abscesses, echinococcal cysts, cystic metastases, and lymphoma can manifest as simple cysts, but these are not usually incidental; there is usually a typical history, and there are usually other clues on the study. In any event, these more commonly cause complex appearing cysts. Patients with splenic abscesses are almost always febrile and symptomatic. Abnormal enhancement patterns suggest malignant etiologies such as metastases, lymphoma, and angiosarcoma. Hypervascular masses are frequently due to benign masses such as hemangiomas, hamartomas, littoral cell angiomas, and lymphangiomas, but hypervascular metastases and lymphoma must be considered. Angiosarcomas are rare, and cause irregular hypervascular masses. The differential diagnosis of multiple small hypovascular masses includes granulomatous disease, metastases, and lymphoma.

Most calcifications are benign, but several morphological forms may be associated with malignancy or infection.

## Workup

Lesions with specific benign features, such as a simple cyst in an asymptomatic patient may be dismissed. Prior imaging and history are the key to evaluating indeterminate lesions such as small hypovascular masses. Stable lesions for one year or greater in patients without cancer are benign; if no prior examinations are available then indeterminate lesions in asymptomatic patients should be followed. If there is a history of lymphoma or other cancer, MRI or PET scan should be considered. The most common indication for percutaneous splenic biopsy is an indeterminate splenic mass in a patient with history of lymphoma or other malignancy [4]. Published guidelines, such as those formulated by the American College of Radiology committee on splenic and nodal findings can be extremely useful in approaching these common lesions [1].
